# Pregnancy in autistic women and social medical considerations: scoping review and meta- synthesis

**DOI:** 10.3389/fpsyt.2023.1222127

**Published:** 2023-10-27

**Authors:** Rosaria Ferrara, Pasquale Ricci, Felice Marco Damato, Leonardo Iovino, Lidia Ricci, Giovanni Cicinelli, Roberta Simeoli, Roberto Keller

**Affiliations:** ^1^Department of Anatomy Histology, Legal Medicine and Orthopaedics, Sapienza University of Rome, Rome, Italy; ^2^“Parthenope” University of Naples, Naples, Italy; ^3^Adult Autism Center, Mental Health Department, Local Health Unit ASL Città di Torino, Turin, Italy; ^4^Department of Humanistic Studies, University of Naples Federico II, Naples, Italy; ^5^Neapolisanit Research and Rehabilitation Center srl, Ottaviano, Italy

**Keywords:** autism, autistic women, social medicine, developmental psychology, neurodivergence

## Abstract

**Introduction:**

This article addresses a topic that has been largely overlooked by scientific literature, namely pregnancy in autistic women. Generally, the issue of sexuality in disability, particularly in disabled women, autistic or otherwise, has been underexplored. However, it is necessary to scientifically investigate this topic to propose adequate social and health policies. Therefore, we chose to conduct a scoping review to answer three main questions: “What does it mean for an autistic woman to be pregnant?”; “How do these two conditions coexist?”; “Are health services prepared to receive this population adequately or does autism become a stigma for pregnant women?”

**Methods:**

We conducted a systematic review and qualitative thematic synthesis following the Preferred Reporting Guidelines for Systematic Reviews and Meta-Analyses on autistic women and pregnancy in the last 10 years.

**Results:**

The studies included in our review are 7, extremely diverse in terms of methodologies and sample sizes. Despite the heterogeneity of samples and methodologies, all research tends to highlight the following results. For autistic women during pregnancy, three areas seem to be the most difficult: sensory issues, mood disorders, and relationships with specialists.

**Discussion:**

Our study found that women with ASD face unique challenges during childbirth that differ from those of neurotypical women. Participants often felt belittled, ignored, and uninformed about the care they received, and being placed at the centre of attention was often seen as negative and hindering rather than positive. However, the research shows us how some “expected” results, such as difficulties in breastfeeding, have been disproven.

## 1. Introduction

Fairly little has been written about pregnancy in autistic women, although this topic should be deepened for several reasons. In this article we will consider Autism Spectrum Disorder (ASD) in the light of the latest paradigm-shifting (1), which consider it a different condition, rather than a deficit. It is important to address this issue as the right to health should be ensured for all equally; however, scientific literature has highlighted that autistic people encounter multiple barriers in accessing health services, in many cases they receive ineffective pharmacological treatments, and very often, professionals do not know how to manage problem behaviors or stereotypical behaviors during routine medical examinations (2–4).

Autism is a neurodevelopmental condition with childhood onset (5) (6) that involves difficulties in verbal and non-verbal communication, restricted and repetitive behaviors, sensory peculiarities, different inflammatoryprocesses1 (7) and executive functions ranging from normal to impaired (8). The prevalence estimate of ASD suggests that men appear to receive a diagnosis more frequently compared to women. However, the latest evidence has underlined a trend of underdiagnosis for the autistic condition in women rather than a lower prevalence (9). Therefore, our in-depth analysis of the topic can have multiple intentions and implications: to learn more about autistic women and their experience of pregnancy, and to train health professionals in the culture of neurodiversity and neurodivergence.

Recent literature has paid little attention to how disabled and/or autistic women experience the entire sphere of sexuality. Indeed, reproduction, menstrual cycle, pregnancy, and maternity management still appear taboo. Moreover, as a result social and health services’ have limited knowledge about these topics (10). Thus, autistic women struggle to open up when they seek health and social services. However, despite communication and sensory difficulties, high functioning autistic (including those previously diagnosed as Asperger’s disorder) often marry or have long-term relationships and sometimes even have children (11). It is not uncommon for some individuals with ASD to become aware of their diagnosis in adulthood (12).

However, while, in the last decades, research on the causes and manifestations of autism has made significant progress, a less explored aspect pertains to the potential interaction between some typical ASD’s symptoms, such sensory abnormalities, and pregnancy in women with this condition. Indeed, experiencing heightened or diminished sensory sensitivity during a particularly delicate moment such as pregnancy may have effects on social interactions, predisposition to engage with others, and consequently, on mood, as some studies in this review indicate (10, 13, 14).

Specifically, individuals with autism often experience sensory hyperresponsivity or hyposensitivity, displaying atypical responses to sensory stimuli from the environment (15). These altered sensory sensitivities may involve one or more of the five senses, namely sight, hearing, smell, touch, and taste, and their intensity can vary from person to person (16). Moreover, some studies in recent years are beginning to hypothesize that sensory aspects may underlie the entire autistic symptomatology (17) (18). Therefore, women with autism who desire to become mothers may face unique and complex challenges due to these sensory characteristics.

Pregnancy is a critical period in a woman’s life, during which her body undergoes a series of physical, hormonal, and psychological transformations (19). These changes might be perceived differently by women with autism due to their atypical sensory responses. Heightened or diminished sensitivity to environmental stimuli could amplify the physical and emotional experiences typical of pregnancy, thereby impacting the entire process.

Sensory alterations in women with autism may manifest in various ways during pregnancy (20). For instance, increased sensitivity to sound could make it challenging to tolerate common noises associated with daily life or medical visits, raising the risk of stress and anxiety for pregnant mothers. Moreover, heightened sensitivity to light or smells could intensify the occurrence of nausea and early pregnancy-related discomforts (21).

Conversely, some women with autism may experience reduced tactile sensitivity, which could influence the perception of typical physical changes during pregnancy, such as sensations related to fetal movements (22). These altered sensory experiences might also impact the pregnant mother’s ability to respond to her body’s needs during pregnancy, leading to reduced awareness of physical changes or potential complications (13).

Furthermore, the way women with autism interact with others during pregnancy may be influenced by sensory aspects (23). Difficulties in understanding non-verbal communication nuances and social interactions could complicate social support during this critical period, influencing the emotional well-being of the expectant mother.

Thus, sensory processing can have a significant impact on childbirth-related tasks such as breastfeeding which could represent a very important dimension due to the bodily and sensory dimensions involved (24).

Understanding the impact of sensory aspects related to ASD on pregnancy is crucial for providing adequate support and assistance to women with autism who wish to become mothers. Clearly, in addition to these sensory challenges, autistic women face difficulties related to communication during interactions with healthcare providers, express a lack of information and appropriate support, and experience distress due to a perception of lack of control during childbirth. Each of these stress factors has negative implications for the mental well-being and emotional state of women during pregnancy and childbirth.

It is also known that, in adulthood, autism is often accompanied by other psychopathologies such as anxiety and depression (25) or also personality disorder or psychosis (26). Pregnancy and motherhood are sensitive transitions for all women, and even more for autistic females who have a greater risk of developing psychopathological comorbidities (27) such as postpartum depression.

Increased awareness of these challenges allows healthcare professionals and social workers to tailor their practices to ensure a more positive and satisfying pregnancy experience for these women. Additionally, research in this field can contribute to the development of targeted interventions to address the specific sensory needs of women with autism during pregnancy, thereby improving their overall well-being and that of their children (28).

## 2. Methods

In this scoping review, we examined the literature covering the issue of autistic women and pregnancy. A scoping review can be a useful tool to explore the landscape of a developing issue. We conducted a scoping review and qualitative synthesis of themes following Preferred Reporting Guidelines for Systematic Reviews and Meta-Analyses [PRISMA; (29)].

The research team searched three databases – PubMed, Psychinfo, and Web of Science – for scientific literature on autistic women and pregnancy in the last 10 years. The research team considered it necessary and sufficient to search these three databases. The review was conducted following the protocol suggested by Arksey and O’Malley (30), specifically designed for individuals with disabilities or similar conditions.

### 2.1. Stage 1: identify the research question

The investigation begins with an overview of the issue and any potential complications. Therefore, we pondered if this issue had received enough attention in the scientific community. What does it mean for an autistic woman to be pregnant? The scientific literature highlights a worsening of some symptoms of autism, especially regarding sensorial issues and mood. How do these two variables interact in pregnant autistic women? Are health services ready to adequately accommodate this population, or does autism become a stigma for pregnant women?

### 2.2. Stage 2: identify relevant studies

To find studies relevant to the given study questions, keywords were used. The following search phrases were used: “Autistic women pregnancy” and “pregnant” or “childbirth.” The eligibility criteria included studies published in the English language between 2013 and 2023. Articles were included if: (a) the sample included individuals with a clinical diagnosis of autism, or Asperger’s Syndrome (AS); (b) participants were facing or had already faced one or more pregnancies.

### 2.3. Stage 3: study selection

**Figure 1:**
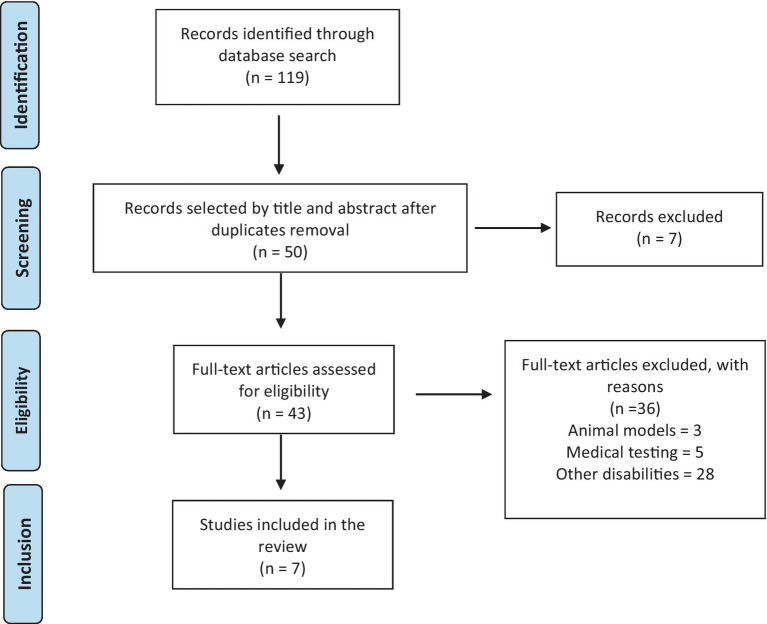
Prisma flow diagram.

The search was conducted through three databases: (i) Psychinfo; (ii) Web of Science; (iii) PubMed. A total of 119 articles were identified, 50 records were selected by title and abstract after duplicates removal, 43 records were assessed as full-text eligible articles. At this step, articles were excluded if: (a) based on animal models; (b) based on medical testing; (c) concerned other disabilities and not specifically autism. The non-specificity of various contributions, which have investigated the issue in the vast sea of disability, is a major limitation of this area of research (Figure 1).

### 2.4. Stage 4: chart the data

The organization of the data from the chosen articles was the fourth stage of the scoping review framework. This stage was carried out using Microsoft Excel. Author(s), title, publication year, the country where the first author’s university is affiliated, research setting, purpose, participant demographics, research methods, measures, interventions, important findings, and limitations were the data points that were gathered Table 1.

**Table 1:** Provides a summary of the authors, participants, measurements, and conclusions.

References	*N*	Methodology	Demographics	Primary findings
Gardner et al. (13)	8	Secondary analysis of a qualitative data set that evolved during the process of developing a research questionnaire to assess childbearing experiences of women with Asperger syndrome.	Eight women (Asperger Syndrome) with average age of 39 years (range, 27–52 years).	Results are presented according to the traditional three stages of pregnancy – the prenatal period, intrapartum period, and postpartum period – which is the model that most clinicians use when providing care. Additionally, women have suggested some potential ideas that may be helpful for clinicians when caring for this population of women.
Rogers (10)	1	Emails correspondence during pregnancy and after. One interview after childbirth.	Melanie, 26 years old.	Melanie’s experience reveals communication difficulties with professionals, and a perception of hospital very “heavy” of sensory elements: noises, shouting, touching and being touched represented a strong stressor for Melanie during visits.
Donovan (31)	24	Interviews conducted using a semi structured interview guide.	Twenty-four women ages 29–65 years from the United States, United Kingdom, and Australia, all of whom gave birth to healthy new-borns in an acute care setting.	Study participants expressed communication difficulties with nurses in a variety of ways. Including trouble conveying needs, alerting nurses when they felt ill, or not understanding what was said to them. Ineffective communication with nurses resulted in feelings of anxiety and being scared and inhibited participants in further attempts at communication.
Phol et al. (33)	487	Online survey and an analysis of the answer using Chi-squared analysis.	Autistic mothers (*n* = 355), and non-autistic mothers (*n* = 132), each of whom had at least one autistic child, were included in our final analysis.	Autistic mothers face unique challenges and the stigma associated with autism may further exacerbate communication difficulties. Greater understanding and acceptance amongst individuals who interact with autistic mothers is needed, and autistic mothers would benefit from additional and better-tailored support.
Lewis et al. (14)	16	Online interviews.	16 autistic women shared 19 birth stories.	Nurses need to provide thorough and nonjudgmental education about the birth process to ensure that autistic women feel safe and in control and do not withdraw from care.
Dugdale et al. (35)	9	Semi-structured interview and narrative analysis.	9 autistic mothers with at least one child between the ages of 5 and 15.	Four themes emerged: 1. *Autism fundamentally impacts parenting*; 2. *Battle for the right support*; 3. *Development and acceptance*; and 4. *The ups and downs of parenting*. The majority of participants’ kids either had an autism diagnosis or were undergoing testing. Subjects also discussed their social and communication problems, such as finding it difficult to socialize, feeling different or having sensory demands.
Hampton et al. (34)	45	Interviews conducted using a semi structured interview guide.	24 autistic and 21 non-autistic women during the third trimester of pregnancy.	The study’s findings suggest that prenatal healthcare for autistic individuals can be improved by adjusting sensory and communication needs. To ensure that autistic individuals receive appropriate support, prenatal healthcare professionals need to receive more comprehensive training related to autism.

### 2.5. Stage 5: collate, summarize, and report results

The final step of the Arksey and O’Malley (30) paradigm involved categorizing the pertinent findings into themes, giving outcomes priority depending on their applicability to the research objectives, and placing a strong emphasis on the type of intervention. Relevant information was provided, such as sample size, participants, procedures, and results. The results section below contains a complete report of all data.

## 3. Results

Jane Donovan (31) conducted a study in 2020 to describe the childbirth experiences of women with ASD. Twenty-four women between the age of 29 and 65 from different countries around the world, who had given birth to healthy newborns, were interviewed. The interviews took place in a setting of their choice, where they felt comfortable. Some chose their home, while others preferred telephone interviews or video calls via Skype/Facebook. Three main themes emerged from the results obtained: communication difficulties, feeling stressed in an uncertain environment and becoming an autistic mother. In conclusion, participants expressed difficulties in communicating with the nurses Communication problems included difficulties in conveying their needs, alerting nurses when they felt unwell, or not understanding what was communicated to them. This ineffective communication resulted in feelings of anxiety and fear and prevented participants from making further attempts to communicate.

Lewis et al. (14), in 2021, conducted a narrative analysis with online interviews. The women who participated had to be over 18 years old, have at least one birth experience, and a self-diagnosis of autism. In this study, 16 self-identified autistic women took part, and two of them shared multiple birth stories, resulting in a total of 19 birth narratives. Participants ages at the time of the study ranged from 21 to 57 years, while their ages at time of giving birth ranged from 19 and 41 years. The interval since giving birth ranged from 6 months to 26 years. All participants identified as female, and most of them were white. Six participants were from the United States, six from the United Kingdom, and two from New Zealand. Five participants were aware of their autism diagnosis at the time of the survey. The narrative method for data analysis used in this study was based on Burke’s (32) dramatistic pentad, which identifies five elements of a story: Act, Scene, Agent, Agency, and Purpose. The most frequent source of trouble (Agency) was an imbalance between a nursing action or a healthcare provider’s act and how that action was carried out. Participants felt that their experiences were minimized, their wishes were ignored, and they were denied important education and communication by those involved in their care due to the actions, comments, and tone of the members of the healthcare team, all of which were frequently perceived as lacking compassion. Many participants believed that they were in labor and required hospital admission, or that their labor had progressed, and needed examination. However, participants admitted that their concerns were belittled by the medical staff, which made them feel uncomfortable. The second most frequent reason for the trouble was an imbalance between the qualities of the person giving birth (Agent) and the environment where the delivery took place (Scene); this problem was frequently linked to autistic characteristics. Participants stated that sensory events related to birth, such as sights, sounds, smells, pressure, and temperature, exacerbated their existing sensory hypersensitivities and caused discomfort, dissociation, and trauma. Many participants reported feeling so overstimulated by their senses that they became dissociated throughout labor, significantly impacting their ability to interact with the medical staff and actively participate in labor. Participants indicated that the social and sensory stimuli in the birthing environment (Scene) as well as how they were handled by medical staff members (Agency) had the most influence on shaping their birth stories.

Pohl and collaborators (33) conducted a study, in 2020, on autistic and non-autistic mothers. 410 autistic mothers and 258 non-autistic mothers participated and completed the survey. Mothers with autistic children were then excluded, reducing the number of non-autistic mothers to 132 and autistic mothers to 355. Two- thirds of the sample of autistic mothers had received the diagnosis, while the remaining third had not. Prenatal and postnatal depression were also substantially more common in autistic moms than in non- autistic mothers. Regarding whether they felt the birthing process had been appropriately described to them, autistic moms were more likely to feel this way than non-autistic mothers. There was also a significant difference between the groups in terms of reporting this. Antenatal class attendance, however, did not change significantly between groups. Autistic mothers were more likely than non-autistic mothers to require multitasking in parenting and housework. They were also more likely to create socialization opportunities for the child and perceive themselves as more organized parents than non-autistic mothers. There were no significant differences between autistic and non-autistic mothers in their ability to put their child’s needs ahead of their own or seek ways to boost their child’s self-confidence. Within the group of mothers with autism, the majority (61%) felt they should have been provided with additional support because of their diagnosis, and 41% of mothers received inadequate support from institutional sources such as hospitals or clinics.

Hampton and collaborators (34) conducted a study on the last trimester of pregnancy involving 24 autistic and 21 non-autistic women. Semi-structured interviews lasting 20 to 60 min were conducted midway through the third trimester of pregnancy. The interviews discussed the bodily and sensory experiences of pregnancy, as well as encounters with medical experts. Nine subthemes were grouped into three main themes: “The physical and psychological impact of pregnancy,” “The influence of official and informal assistance,” and “Fears and hopes of parenthood.”

While the non-autistic group reported only sensory changes related to smell and taste, the autistic group frequently mentioned changes in sound, lighting, and touch. The participants in the autistic group also reported feeling more anxious and depressed with some of them connecting these changes to hormonal factors. Both groups discussed physical exhaustion, but several members of the autistic group also discussed mental exhaustion and related issues. Furthermore, the group of autistic women clearly expressed their preference for specialist-patient communication in writing or by telephone. Furthermore, autistic women expressed concern about the hospital environment, which they considered inadequate and lacking in training to accommodate autistic women. They also expressed concerns about the executive functions required for parenthood.

Dugdale and collaborators (35) conducted research on 9 autistic mothers with at least one child between the ages of 5 and 15. The women were asked to complete a semi-structured interview which was subsequently analyzed from a narrative point of view. Four main themes emerged: (i) Autism fundamentally impacts parenting; (ii) Battle for the right support; (iii) Development and acceptance; and (iv) The ups and downs of parenting. Most participants’ children either had an autism diagnosis or were undergoing testing. The participants also discussed their social and communication challenges, such as finding it difficult to “socialize with other parents,” feeling different or having sensory demands. Shared diagnoses helped participants feel closer and more connected to their children. Another frequent challenge was managing sensory sensitivity while parenting, particularly during pregnancy. Participants described difficulties in seeking help for themselves or their children because they felt misunderstood, judged, or disregarded. Participants discussed how having an autistic trait was often associated with being misunderstood. All the participants received an autism diagnosis after becoming parents. Some interviewees reported feeling “guilty” before receiving a diagnosis. Before their diagnosis, some participants spoke of feeling “sorry” for their struggles and how they affected their children. After receiving a diagnosis, participants’ experiences were positively “re-process[ed].” Many found that this lessened their sense of guilt, improved their self-acceptance, and provided an explanation that was consistent with who they were. The “hardest thing” about parenting, according to many, was dealing with their children’s autism or other special needs. Some participants believed this was because their child was “different,” and they felt they did not belong in “the usual mom’s club.”

Rogers and collaborators (10) conducted a case study and shared the findings obtained from the stories of Melanie, a girl with Asperger. Melanie’s story was elicited through emails—pre and post birth, and through one interview (post birth). She was very eager to participate with the researchers and to share her story. The interview was audio recorded, transcribed, and verified by the participant. Three themes emerged from the thematic analysis of Melanie’s narrative, the interview, and the content of her emails. The main theme related to the communication and services issues she encountered with healthcare professionals. Sensory issues were also very prominent in her narrative, and the challenges of parenting were quite evident. Melanie first contacted the researchers by email when she was 6 months’ pregnant. Her narrative began with a concise description of her childhood. At 25 weeks, Melanie started to make more frequent visits to the hospital where her sensory stress became more pronounced. The girl described some of these experiences in detail. Melanie’s account reveals her perceptions of how ASD affected her perinatal experience, as well as her belief that ASD influenced the way her midwives and doctors conducted their medical assessments. Furthermore, the young woman highlighted how even the hospital and its staff were too very “heavy” sensory elements: noises, shouting, touching and being touched represented a strong stressor for Melanie.

Marcia Gardner and collaborators (13) conducted a study on the pregnancy experience of women with Asperger’s syndrome (ASD). This qualitative study describes the childbearing experiences of eight women with Asperger syndrome. Four major themes emerged: Processing Sensations, Needing to Have Control, Walking in the Dark, and Motherhood on My Own Terms. Most women commented about difficulties in processing sensations associated with pregnancy and heightened sensitivities to touch, light, sounds, and interaction. Additionally, several women felt they had less control over their actions and environment. Deciphering the child’s behavior was also a challenging task, and the women received help from their mothers. The feeling of judgment and expectations toward them came from the medical-health environment and the professionals they met.

## 4. Discussion

The selected studies in this review appear to share some results and limitations, the latter primarily related to heterogeneous methodologies and samples.

The diagnosis of autism in adulthood still appears to be one of the major clinical challenges to overcome, and it seems to be even more difficult if requested by a woman. Much has been discussed about the camouflage effect of autism in autistic women, which means a greater cognitive ability of women to hide the signs and symptoms of autism. Therefore, many women participating in autism research have not received a formal diagnosis, but have self-diagnosis, leading them to the awareness of being on the autism spectrum. Currently, researchers are forced to accept self-diagnoses of autism; otherwise, there would be a risk of not having enough subjects to be involved. However, this limitation must be taken into consideration.

Regarding our review goals, the consulted research has led us to highlight the following results:

Regarding aspects related to the sensory sphere, the results have shown that pregnancy for autistic women represents a moment with more sensory challenges compared to non-autistic women. In fact, while sensory changes in the non-autistic group were limited to smell and taste, the autistic group commonly reported changes involving sound, light, and touch, making everyday life more difficult. Therefore, aspects related to abnormal sensory perception were inevitably linked to both relational and emotional difficulties (14, 34).

From an emotional perspective, it emerged that the group of autistic women showed an increase in anxiety and low mood (31). Additionally, both groups stated that being pregnant attracted social attention, and for neurotypical women, this situation tended to be pleasant, while autistic women found it difficult to manage an increase in conversations and being the center of attention (35).

Finally, in line with our hypotheses, the different way in which people with autism perceive stimuli determines not only different sensory experiences but also distinct emotional experiences, which medical specialists (e.g., gynecologists and midwives) often struggle to understand and effectively respond to the needs and requests of their patients during that time (10). Our studies have highlighted that, autistic mothers experience greater difficulty in interacting with professionals during their pregnancy; they prefer to communicate with professionals via a phone call rather than in person. Moreover, for many women, discussing their autism during pregnancy represents more of a stigma than a source of support. In fact, the prevailing belief among women is that revealing their autism would lead specialists to treat them worse, which also highlights a certain fear of being judged by others.

Therefore, the examined studies have found that women with ASD face unique challenges during childbirth that differ from those of neurotypical women. It emerged that the participants often felt belittled, ignored, and uninformed about the care they received, also experiencing being at the center of attention as something negative and hindering, rather than positive. On the other hand, the research shows us how some “expected” results, such as difficulties in breastfeeding, have been contradicted (autistic women may experience slightly more discomfort in breastfeeding but still prefer it because it is considered “better” for the child). Meanwhile, there seems to be an urgent need to refine specific identification and intervention protocols for autism in adulthood. The current situation presents us with professionals who are not prepared to handle the specific communicative, physical, emotional, and sensory needs of autistic women, thereby they are unable to provide adequate assistance.

One of the limitations of this review is certainly related to the scarcity of articles in the literature on the topic of pregnancy among autistic women, indicating the limited attention given to this topic and the consequent availability of knowledge in clinical settings.

## 5. Conclusion

Pregnancy can be a challenging experience for any woman, bringing both joy and a multitude of physical and physiological changes. However, for autistic women, the changes that come with pregnancy can be particularly overwhelming and confusing. During pregnancy, in fact, autistic women may experience difficulties with sensory processing, depression, anxiety, and fatigue, making it challenging to carry out the day-by-day activities required for a healthy pregnancy. To address these challenges, a medical and social approach could be adopted to provide support for autistic women during this delicate time. On the other hand, professionals should be trained on autism to address the impact of Autism during pregnancy. The deepening of this topic could also contribute to that movement of increasingly in-depth analysis of the autistic phenotype and its various expressions (36).

In this regard, the National Autistic Society has proposed the use of a “Health Passport” to help autistic individuals communicate their needs to healthcare professionals, including doctors and nurses. Widespread use of this tool, along with extensive training for healthcare personnel on managing the needs of pregnant autistic women, with a focus on communication and sensory aspects, could be particularly beneficial. In summary, identifying the unique challenges faced by pregnant autistic women and providing appropriate support could go a long way in ensuring their well-being during this critical period. Services for ASD should propose programs for ASD individuals and caregivers concerning social skill training to improve abilities in relationships and affective-sexual training programs.

To address this issue in our Adult Autism Center, we propose a specific sexual education program for people with autism in adulthood. The aim is to improve knowledge of the body and enhance social skills to form relationships. The program has three different levels, related to the same level of ASD described in DSM 5 (6). The teaching team is composed of psychologists, psychiatrists, urologists, and gynecologists. All the teachers are also specialized in autism and disability. The course is gender-mixed, involving males and females in the same group. In the ASD level-1 course, only ASD individuals are admitted. In the ASD level-2 course, ASD individuals may be supported during the lessons by their social coach, who usually assists them in daily activities. The ASD level-3 course is directed to patients’ caregivers. Topics taught include body anatomy, contraception, coitus, and pregnancy. In a separate program, a social-skill training program is focused on improving social abilities. ASD individuals should be prepared for these issues.

## Data availability statement

The original contributions presented in the study are included in the article/supplementary material, further inquiries can be directed to the corresponding author.

## Author contributions

RF, PR, and GC contributed to the conception and design of the study. RF wrote the first draft of the manuscript. LR and FD organized the database and performed the statistical analysis. RK and RS reviewed all the papers. All authors contributed to the article and approved the submitted version.
